# Chemotherapy induces ACE2 expression in breast cancer via the ROS-AKT-HIF-1α signaling pathway: a potential prognostic marker for breast cancer patients receiving chemotherapy

**DOI:** 10.1186/s12967-022-03716-w

**Published:** 2022-11-05

**Authors:** Xiaoyan Zuo, Sixin Ren, He Zhang, Jianfei Tian, Ruinan Tian, Baoai Han, Hui Liu, Qian Dong, Zhiyong Wang, Yanfen Cui, Ruifang Niu, Fei Zhang

**Affiliations:** 1 Public Laboratory, Tianjin Medical University Cancer Institute and Hospital, National Clinical Research Center for Cancer, Tianjin, 300060 China; 2 Key Laboratory of Cancer Prevention and Therapy, 300060 Tianjin, China; 3 Tianjin’s Clinical Research Center for Cancer, 300060 Tianjin, China; 4 Key Laboratory of Breast Cancer Prevention and Therapy, Tianjin Medical University, Ministry of Education, Tianjin, 300060 China

**Keywords:** Angiotensin-converting enzyme 2 (ACE2), Biomarker, ROS, Chemotherapy resistance, Prognosis, Breast cancer

## Abstract

**Background:**

Angiotensin-converting enzyme 2 (ACE2) is a key enzyme of the renin-angiotensin system and a well-known functional receptor for the entry of severe acute respiratory syndrome coronavirus 2 (SARS-CoV-2) into host cells. The COVID-19 pandemic has brought ACE2 into the spotlight, and ACE2 expression in tumors and its relationship with SARS-COV-2 infection and prognosis of cancer patients have received extensive attention. However, the association between ACE2 expression and tumor therapy and prognosis, especially in breast cancer, remains ambiguous and requires further investigation. We have previously reported that ACE2 is elevated in drug-resistant breast cancer cells, but the exact function of ACE2 in drug resistance and progression of this malignant disease has not been explored.

**Methods:**

The expression of ACE2 and HIF-1α in parental and drug-resistant breast cancer cells under normoxic and hypoxic conditions was analyzed by Western blot and qRT-PCR methods. The protein levels of ACE2 in plasma samples from breast cancer patients were examined by ELISA. The relationship between ACE2 expression and breast cancer treatment and prognosis was analyzed using clinical specimens and public databases. The reactive oxygen species (ROS) levels in breast cancer cells were measured by using a fluorescent probe. Small interfering RNAs (siRNAs) or lentivirus-mediated shRNA was used to silence ACE2 and HIF-1α expression in cellular models. The effect of ACE2 knockdown on drug resistance in breast cancer was determined by Cell Counting Kit 8 (CCK-8)-based assay, colony formation assay, apoptosis and EdU assay.

**Results:**

ACE2 expression is relatively low in breast cancer cells, but increases rapidly and specifically after exposure to anticancer drugs, and remains high after resistance is acquired. Mechanistically, chemotherapeutic agents increase ACE2 expression in breast cancer cells by inducing intracellular ROS production, and increased ROS levels enhance AKT phosphorylation and subsequently increase HIF-1α expression, which in turn upregulates ACE2 expression. Although ACE2 levels in plasma and cancer tissues are lower in breast cancer patients compared with healthy controls, elevated ACE2 in patients after chemotherapy is a predictor of poor treatment response and an unfavorable prognostic factor for survival in breast cancer patients.

**Conclusion:**

ACE2 is a gene in breast cancer cells that responds rapidly to chemotherapeutic agents through the ROS-AKT-HIF-1α axis. Elevated ACE2 modulates the sensitivity of breast cancer cells to anticancer drugs by optimizing the balance of intracellular ROS. Moreover, increased ACE2 is not only a predictor of poor response to chemotherapy, but is also associated with a worse prognosis in breast cancer patients. Thus, our findings provide novel insights into the spatiotemporal differences in the function of ACE2 in the initiation and progression of breast cancer.

**Supplementary Information:**

The online version contains supplementary material available at 10.1186/s12967-022-03716-w.

## Introduction

As the key functional receptor for severe acute respiratory syndrome coronavirus 2 (SARS-CoV-2) entry into host cells [1], the global pandemic of COVID-19 has brought angiotensin-converting enzyme 2 (ACE2) into the spotlight. ACE2, a zinc metallopeptidase, is a major regulatory enzyme of the vascular protective axis of the renin-angiotensin system (RAS) [2]. The activation of ACE2 catalyzes the production of Angiotensin-(1–7) (Ang-(1–7)) from the peptide hormone Angiotensin-II (Ang-II), which is generated by ACE cleavage of the C-terminus of Ang-I [2]. Generally, the binding of Ang-II to its receptor AT1 acts to constrict blood vessels to increase blood pressure, as well as activates and amplifies the inflammatory response [3]. Conversely, Ang-(1–7) exerts vasodilatory, anti-inflammatory, anti-proliferative and anti-fibrotic effects by binding to its receptor MasR [2, 4–6]. Therefore, the ACE2/Ang-(1–7)/MasR signal pathway physiologically functions as a negative modulator of the classical ACE/Ang-II/AT1R axis [7]. The balance between these two axes of the RAS is critical for maintaining cardiovascular homeostasis, brain and renal function [8–10]. Dysregulation of the RAS is associated with a variety of diseases, including hypertension, atherosclerosis, diabetic nephropathy and even malignancies [11–14]. However, the exact function and mechanism of ACE/ACE2 in the development and progression of these diseases remain unclear and require further investigation.

Altered expression of ACE or ACE2 is associated with initiation and progression of many cancers. In most cases, elevated expression of ACE and its downstream receptor AT1R is related with adverse characteristics and poor outcomes in cancer patients [15]. Consistently, the activation of ACE/Ang-II/AT1R axis promotes proliferation, invasion, angiogenesis, epithelial-mesenchymal transition (EMT), and resistance in several cancer models [15–17]. In addition, ACE-targeted inhibitors have been used in preclinical trials with some promising results [18]. In contrast, ACE2 expression is decreased in most types of cancer tissue, and reduced ACE2 is often associated with a poorer prognosis [19–21]. Functional studies have found that the activation of ACE2/Ang-(1–7)/MasR axis can inhibit proliferation, angiogenesis, invasion and metastasis of cancer cells, thereby counteracting the biological effects of the ACE/Ang II system [19, 22, 23]. Nevertheless, not all reports consider ACE2 as a tumor suppressor. Notably, several studies have shown that upregulation of ACE2 is a marker of poor prognosis in some cancer patients [24, 25]. Thus, the function of ACE2 may be multifaceted, and its specific role in tumors may depend on tissue type and context.

Recently, several papers have reported the correlation between ACE2 expression and the prognosis of breast cancer. Overall, ACE2 expression was significantly lower in tumor tissues than in normal tissues, and patients with higher ACE2 expression have a favorable outcome [26–28]. In addition, in vitro studies found that ACE2 upregulation inhibited the invasion and metastasis of breast cancer cells and vice versa [26, 29]. Hence, ACE2 appears to act a tumor suppressor in breast cancer. However, there are different views on the relationship between ACE2 expression and patient prognosis. A recent study reported inconsistent results that ACE2 upregulation is a marker of poor prognosis in HER2 subtype breast cancer [24]. It is well known that the same signaling pathway may play different roles in different cellular contexts. Thus, the effect of ACE2 on cancer progression varies by tumor entity or subtype. Taken together, these findings suggest that the biological effect of ACE2 on breast cancer progression may be subtypical and stage-specific.

Considering the critical role of ACE2 in tumorigenesis and progression, the mechanisms that regulate ACE2 expression have received much attention. Recent studies have found that several external stimuli, such as SARS-COV-2 infection and smoking, can upregulate the expression of ACE2 in respiratory epithelial cells. In addition, inflammation-related signaling pathways can also result in the upregulation of ACE2 in some epithelial cells [30–33]. These results suggest that the mechanisms regulating ACE2 expression are complex and diverse, and may be related to cell type and stimuli. To date, the regulatory mechanism of altered ACE2 expression in cancer cells remains unclear. In addition, few previous studies on breast cancer have addressed the correlation between ACE2 expression and treatment response. These issues should be clarified. In the present study, we investigated the relationship between ACE2 expression and treatment resistance and prognosis of breast cancer. We identified a potential mechanism by which chemotherapeutic agents induce ACE2 expression in breast cancer cells. We explored the role of elevated ACE2 in predicting chemotherapy response and prognosis in breast cancer patients.

## Materials and methods

### Reagents and drugs

RPMI 1640 and DMEM/F12 medium, Fetal bovine serum (FBS) and Trypsin for cell culture were obtained from Hyclone (Logan, UT, USA). Drugs used in this study are as follows: Epirubicin (EPI) was obtained from Hanhui Pharmaceuticals (Jiangsu, China). 5-Fluorouracil (5-FU) was purchased from Sigma. Paclitaxel (PTX) was obtained from Aosaikang Pharmaceuticals (Zhejiang, China). The AKT inhibitor MK-2206 (HY-10358) and HIF-1α inhibitor YC-1 (HY-14927) were purchased from MCE. Cell counting kit-8 (CCK8) was obtained from Bimake (Houston, TX, USA). HiScript II Q RT SuperMix for qPCR and Ace qPCR SYBR Green Master Mix were obtained from Vazyme (Nanjing, China). Trizol reagent was purchased from Invitrogen (Carlsbad, CA, USA). Enhanced chemiluminescence (ECL) was performed using ECL kit (Bio-Rad). The antibodies are listed as follows: ACE2 (ab108252) and HIF-1α (ab51608) were purchased from Abcam (Cambridge, MA, USA), AKT (#9272) and phospho-Akt (Thr308 #4370s) were obtained from Cell Signaling Technology (CST, Beverly, MA, USA), and β-actin was purchased from Sigma-Aldrich (St. Louis, MO, USA). These antibodies were diluted in 5% BSA, the diluted ratio of ACE2 was 1:2000, HIF-1α was 1:1000, AKT and Phospho-Akt (Thr308) were 1:2500, and β-actin was 1:5000.

### Cell culture

Human breast cancer cell lines (MDA-MB-468, SK-BR-3, MDA-MB-231, BT-474, T47D and MCF-7), colorectal cancer cell lines (SW620 and SW480) and pancreatic cancer cell lines (SU86.86 and SW1990) were purchased from American Type Culture Collection (ATCC). Chemoresistant cancer cell lines SK-BR-3/Epirubicin (SK/EPR), MDA-MB-468/Epirubicin (468/EPR), MDA-MB-468/Paclitaxel (468/PTR) and BT-474/Lapatinib (BT-474/Lapa) were previously established by our group [34, 35]. MDA-MB-468, 468/EPR and 468/PTR cells were cultured in DMEM/F12 medium, other cells were cultured in RPMI-1640 medium, and all medium contains 10% fetal bovine serum. All the cells were maintained in an incubator containing 5% CO_2_ at 37 °C.

### Western blotting

Western blotting assay was carried out as previously described [36]. Briefly, cultured cells in the dish were washed with ice-cold phosphate-buffered saline and then lysed in cell lysis buffer (50 mM Tris, 150 mM NaCl, 2% SDS, 10% glycerol, 5% 2-mercaptoethanol, and 1× protease inhibitor cocktail at pH 6.8) on ice. After 30 min, the cell lysates were collected, boiled at 95 ℃ for 10 min, and then resolved by SDS–PAGE using a 10% gel. Subsequently, the proteins were transferred onto a polyvinylidene fluoride membrane (Millipore). The membranes were blocked with 5% nonfat milk in TBST, and incubated with primary antibodies overnight at 4 ℃, followed by detection with horseradish peroxidase-conjugated secondary antibodies. The protein bands were detected by chemiluminescence using an ECL kit. β-actin was used as a loading control.

### Quantitative reverse transcription PCR (qRT-PCR)

Quantitative reverse transcription PCR (qRT-PCR) analysis was performed with a AceQ qPCR SYBR Green Master Mix (Vazyme, China) as previously described [37]. In brief, cells treated with different doses of drugs (EPI, PTX and 5-FU) were lysed in Trizol, then total RNA were extracted according to the manufacturer’s instructions and reverse transcribed into cDNA by HiScript II Q RT SuperMix for qPCR (Vazyme, China). The qPCR was performed using AceQ qPCR SYBR Green Master Mix (Vazyme, China) following the manufacturer’s protocol. For analyzing relative changes in gene expression, the 2^− ΔΔct^ method was used. The expression levels of ACE2 and HIF-1α were normalized to β-actin. The primer sequences were shown in Additional file 1: Table S1.

### siRNA and cell transfection

Small interference RNAs (siRNAs) specifically targeting ACE2 or HIF-1α mRNA were synthesized by GenePharma (Tianjin, China). The siRNAs were transfected into the cells cultured in 6-well plates using lipofectamine RNAiMax (Thermal Fisher Scientific) following the manufacturer’s instructions. The sequences of the siRNAs are shown in Additional file 1: Table S2.

### IC_50_ assay

The IC_50_ assay was performed as described previously [34]. In brief, cells were seeded in 96-wells plate at a density of 6 × 10^3^ cells per well for 24 h. Then, the cells were cultured for 48 h in the presence of different concentrations of EPI (0, 0.5, 1, 2, 4, 8, 16, 32, 64, 128, and 256 µM). CCK-8 reagent was added into each well (10 µL/well) and the plate was incubated for additional 3 h at 37 °C. The cell viability was calculated by using a micro-ELISA reader with a 450 nm filter. The half-maximal inhibitory concentration (IC_50_) was calculated based on the relative survival curve using the GraphPad Prism v. 8.0.

### Cell proliferation assay and colony formation assay

The CCK-8 and colony formation assay were used to evaluate cell proliferative activity. For CCK-8 assay, 1 × 10^3^ cells per well were seeded in 96-well plates and incubated for 24 h, 48 h, 72 h, 96 and 120 h. Then, 10 µL of CCK-8 solution was added to each well and incubated for another 3 h in the incubator. Finally, the optical density (OD) value was read at 450 nm using a 96-well plate reader. Colony formation assay was carried out as described previously [36]. In brief, 1 × 10^3^ cells were seeded in 6-well plates and cultured for 10–14 days. Then the cells were washed with PBS, fixed with formaldehyde and stained using crystal violet staining buffer. The number of colonies was counted under an inverted microscope. EdU incorporation assay was performed according to the previous method [36]. In brief, cells in a 96-well plate were cultured overnight, then 10 µM EdU solution was added to the medium, and the cells were incubated for 2 h before measurement. Afterwards, the cells were fixed with 4% paraformaldehyde for 15 min and permeabilized with 0.3% Triton X-100 for another 15 min. Then, the cells were incubated with the Click Reaction Mixture for 30 min at room temperature in dark, followed by incubation with Hoechst 33342 for 10 min to stain the nuclei. Finally, the percentage of EdU-positive cells was used to determine the cell proliferation activity. Apoptosis assay was performed using an Annexin V-FITC/PI Apoptosis Detection Kit (Vazyme, China). Briefly, cells in 6-well plates were treated with EPI for 72 h, then the cells were digested with trypsin, washed with PBS, and resuspended in 300 µL of binding buffer. Subsequently, the resuspended cells were stained with 5 µl of FITC-conjugated Annexin V and 5 µl of PI according to the manufacturer’s instruction, followed by flow cytometric analysis with the FITC and PE channels.

### Measurement of intracellular ROS

Intracellular ROS levels were measured using a Reactive Oxygen Species Assay Kit (Beyotime, China) in accordance with the manufacturer’s instructions. In brief, cells were seeded in 6-well plates, treated with EPI for 72 h, then washed with PBS and incubated with DCFH-DA ROS probe (diluted 1:1000 in serum-free medium) for 20 min at 37 ℃. Afterwards, the cells were trypsinized and washed, and then the stained cells were analyzed by a flow cytometer using the FITC channel. Cells treated with H_2_O_2_ (100 µM) or ROS scavenger NAC (500 µM) were used as positive and negative controls, respectively.

### Construction of ACE2 stable knockdown cell line

ACE2 stable knockdown cell line was constructed using a lentiviral system as described below. The ACE2 shRNA oligo DNAs were designed, synthesized, and cloned into the Age I and BamH I cloning sites of the lentiviral vector pLKO.1-hygromycin to construct the pLKO.1-shACE2 plasmids (Additional file 1: Table S3). The pLKO.1-hygromycin vector expressing scrambled shRNA was used as a negative control (shControl). The lentiviral plasmids and packaging plasmids were co-transfected into HEK293T cells to generate lentivirus. The virus in the medium was collected 48 and 72 h after transfection. Afterward, the drug-resistant breast cancer cell line MDA-MB-468/EPR cells were infected with lentivirus overnight and further selected by using Hygromycin B (200 µg/mL).

### Data resource

The publicly available TCGA-BRCA (http://portal.gdc.cancer.gov/) database and the Molecular Taxonomy of Breast Cancer International Consortium (METABRIC) cohort data (http://www.cbioportal.org/) were used to determine the mRNA expression of ACE2, and we conducted a systematic search in the GEO (https://www.ncbi.nlm.nih.gov/gds) database to identify breast cancer drug-resistant gene expression datasets. The independent cohorts analysis was performed by the online tool named GEO2R (www.ncbi.nlm.nih.gov/geo/geo2r). To more systematically investigate ACE2 expression in breast cancer, gene expression normalized to RPKM (Reads Per Kilobase Million) for breast cancer cell lines (n = 57) was downloaded from the Cancer Cell Line Encyclopedia (CCLE) (https://portals.broadinstitute.org/ccle). We also obtained a set of genes associated with chemoresistance of breast cancer in Genecards database (https://www.genecards.org/).

### Bioinformatics analysis

GSCALite [38] (http://bioinfo.life.hust.edu.cn/web/GSCALite/), a web-based analysis platform for gene set cancer analysis, was performed to evaluate the association of a 28 genes signature (named BRCA-DRGs) with drug sensitivity to validate the efficacy of this genes signature in predicting drug sensitivity. We used the Gene Expression Profiling Interactive Analysis (GEPIA) (http://gepia.cancer-pku.cn/) database to analyze differential ACE2 expression in breast cancer. The freely available online tool EVenn (http://www.ehbio.com/test/venn/) was employed to explore and generate Venn diagrams of five sets. As for survival analysis, we analyzed the relationship of ACE2 expression with relapse-free survival (RFS) and distant metastasis-free survival (DMFS) in breast cancer (BRCA) using Kaplan-Meier Plotter (https://kmplot.com/analysis/).

### Patients and plasma samples

Plasma samples were collected from 111 BRCA patients, including 57 patients who received chemotherapy or neoadjuvant therapy, 54 patients who did not receive chemotherapy, and 20 age-matched healthy controls. All samples were obtained at the Tianjin Medical University Cancer Institute and Hospital (China) between December 2020 and January 2021. Clinical data including sex, age, TNM stage, recurrence and surgery were collected (Additional file 1: Table S4). This study was approved by the Ethics Committee of Tianjin Medical University Cancer Institute and Hospital. Written informed consent was obtained from all participants.

### Enzyme-linked immunosorbent assays (Elisa) for human ACE2

Plasma ACE2 levels were measured with the Elabscience #E-EL-H0281c ELISA kit following the manufacturer’s protocol. 96-well ELISA plates were previously coated with anti-human ACE2 antibodies. Briefly, 100 µL of standard or plasma sample were added into each well and incubated for 90 min at 37 °C. Then, the liquid was discarded and incubated with 100 µL of biotinylated detection antibodies for 60 min at 37 °C. After aspirating and washing the plate three times, 100 µL of HRP conjugate working solution was added and further incubated for 30 min at 37 °C. After another three times washing, the substrate reagent was added (90 µL/well), and the plate was incubated in the dark at room temperature for 15 min. The reaction was stopped with 50 µL/well of stop solution, and the absorbance at 450 nm was measured using an ELISA reader.

### Statistical analysis and visualizations

Statistical software R (version 3.4.1) and GraphPad Prism (version 8.0) were used for statistical analysis. R packages “ggplot2”, “ggrepel”, “ggpubr”, “corrplot” and “grid” were used for data visualization [39]. One-way or two-way ANOVA were used to compare the statistical significance between different groups. For all analyses, *P* < 0.05 was considered statistically significant. All the data were presented as mean ± SD.

## Results

### ACE2 is specifically upregulated in drug-resistant breast cancer cells

**Fig. 1:**
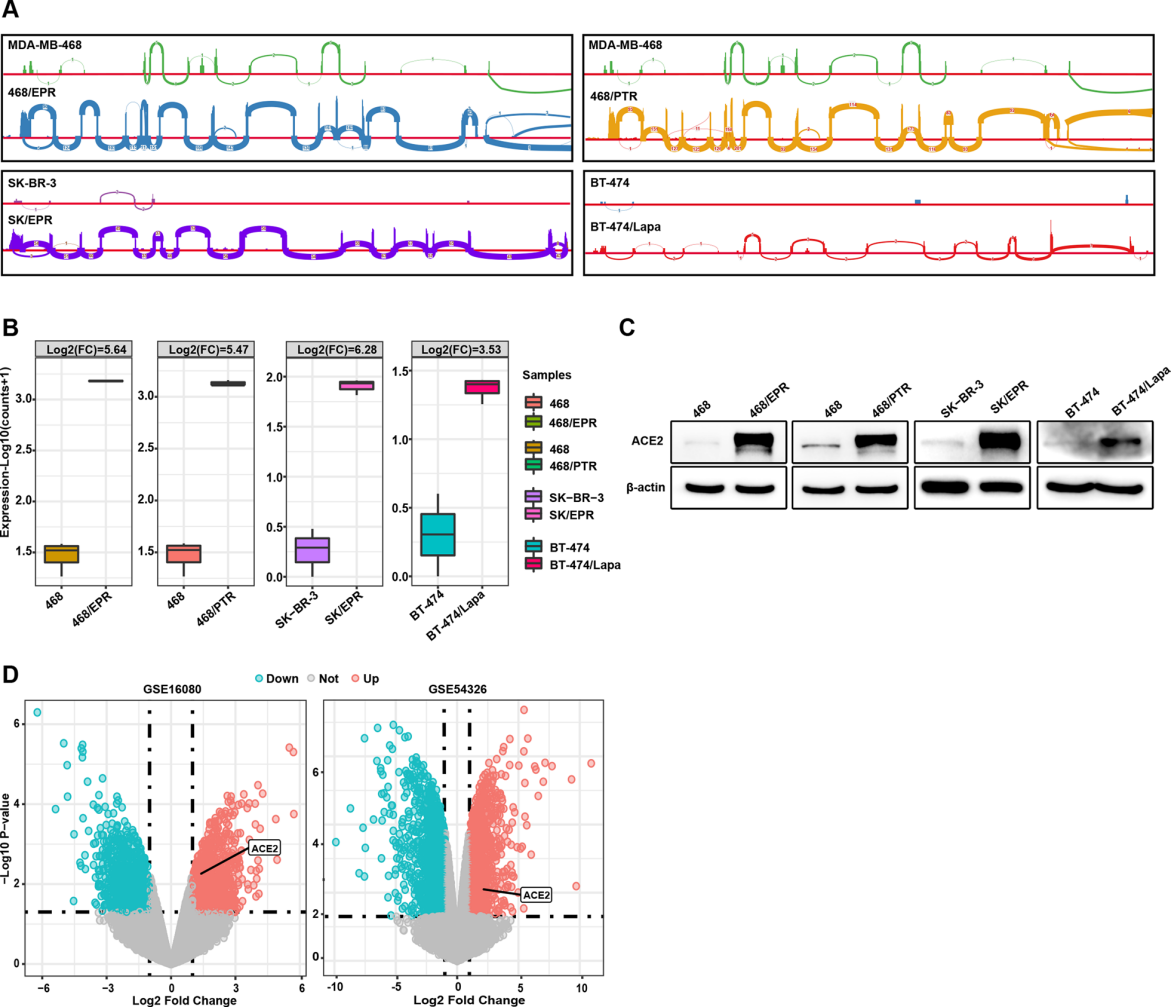
ACE2 is highly expressed in drug-resistant breast cancer cells. **A** Integrative Genomics Viewer (IGV) results showed that drug-resistant cells have substantially more reads mapped onto ACE2 exons compared with the parental cells. **B** Facet boxplot showing upregulation of ACE2 expression in drug-resistant breast cancer cells (*P* < 0.05, LogFC > 1). **C** Western blot analysis of high expression of ACE2 protein in 468/EPR, 468/PTR, SK-BR-3/EPR and BT-474/Lapa cells. **D** Volcano plot showing differentially expressed genes in 468/MTX and SK-BR-3/EPR cells, with labels indicating ACE2 overexpressed in these cells (*P* < 0.05, LogFC > 1).

We previously showed that ACE2 is elevated in EPI-resistant breast cancer MDA-MB-468/EPR cells [35]. To investigate whether elevated ACE2 expression in resistant breast cancer cells is a common phenomenon, we analyzed transcriptome-sequencing data from several parental and resistant cells. These cells resistant to chemotherapeutics and targeted drugs (named as 468/EPR, 468/PTR, SK-BR-3/EPR and BT-474/Lapa) were previously established and sequenced by our group [34, 35]. We first analyzed the RAW data of RNA-seq, which showed that drug-resistant cells had more reads mapping to exons of ACE2 than parental cells, suggesting that ACE2 may be upregulated in drug-resistant breast cancer cells (Fig. 1A). Indeed, as shown in boxplot, the mRNA expression of ACE2 was significantly upregulated in drug-resistant breast cancer cells (LogFC > 1, FDR < 0.05, Fig. 1B). Consistently, Western blot analysis also confirmed that ACE2 expression was substantially increased in the drug-resistant cells compared with parental cells (Fig. 1C). In addition, ACE2 is also drastically elevated in the methotrexate-resistant MDA-MB-468 cell line and the EPI-resistant SK-BR-3 cell line by analyzing two independent cohorts GSE16080 [40] and GSE54326 [41] using the Gene Expression Omnibus (GEO) database. Collectively, these data indicate that the upregulation of ACE2 is a common phenomenon in drug-resistant breast cancer cells.

### The high expression of ACE2 is associated with drug resistance in breast cancer patients

**Fig. 2:**
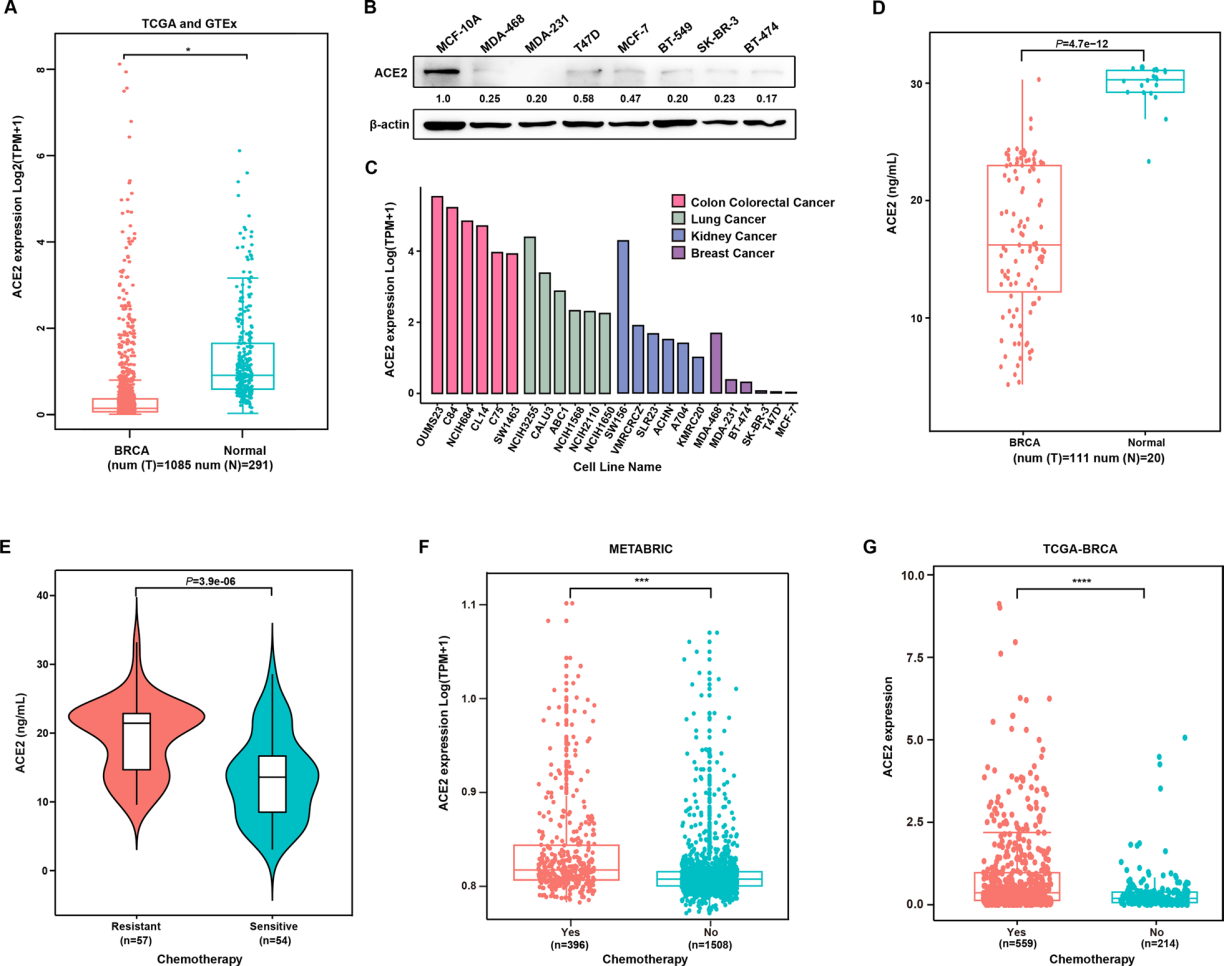
High expression of ACE2 is associated with drug resistance in breast cancer patients. **A** The ACE2 expression profiles of breast cancer samples and normal tissues in the TCGA-BRCA and GTEx database. (Dot plot) Each dot represents the expression of a sample (*P < 0.05). **B** Western blot analysis showed the expression of ACE2 protein in normal breast cell line (MCF-10A) and breast cancer cell lines (MDA-468, MDA-231, T47D, MCF-7, BT-549, SK-BR-3, BT-474). The number below every lane represents the ratio of ACE2/β-actin expression in the Western blot assays. **C** Expression levels of ACE2 gene in breast cancer, colon colorectal cancer, lung cancer, kidney cancer and pancreatic cancer cell lines from the Cancer Cell Line Encyclopedia (CCLE). The vertical axis in the figure represents ACE2 expression, the horizontal axis is the cell lines, and the different colors represent the group of cell lines. **D** ACE2 expression levels in the plasma of all breast cancer patients (n = 111) and healthy controls (n = 20) were measured by ELISA (*P* = 4.7e−12). **E** The plasma levels of ACE2 in chemosensitive and insensitive breast cancer patients (*P* = 3.9e-06). (F) Comparison of ACE2 expression levels in breast cancer patients receiving and not receiving chemotherapy in METABRIC (***P < 0.001) and (G) TCGA-BRCA database (****P < 0.0001)

Previous studies have demonstrated that the expression of ACE2 is reduced in breast cancer tissues compared to normal tissues. Consistently, the result based on the analysis of the TCGA-BRCA and GTEx database showed that the expression of ACE2 in normal tissues was higher than that in breast cancer patients (Fig. 2A). Moreover, we compared the protein expression of ACE2 between breast cancer cells and human normal breast epithelial cells MCF-10 A using western blot assay. As shown in Fig. 2B, breast cancer cells indeed expressed lower levels of ACE2 compared to normal breast cell MCF-10A. In addition, as revealed by CCLE analysis, the expression level of ACE2 was lower in breast cancer cell lines than in other cancer cell lines, including colorectal, lung, and kidney cancers (Fig. 2C). To further determine the role of ACE2 in breast cancer, we collected plasma samples from breast cancer patients and healthy donors and measured the level of ACE2 by ELISA. As shown in Fig. 2D, the overall level of ACE2 in the plasma of breast cancer patients is still lower than that of healthy control. Intriguingly, when classifying breast cancer patients according to their sensitivity to chemotherapy, we found that plasma ACE2 levels were significantly higher in chemotherapy-insensitive patients than in sensitive patients (Fig. 2E; Table 1, *P <* 0.0001). In agreement with this finding, we also observed a similar association between upregulation of ACE2 levels and sensitivity to chemotherapy in breast cancer patients in the METABRIC and TCGA-BRCA database (Fig. 2F and G). Altogether, these results indicate that although ACE2 expression is decreased in breast cancer patients compared with the normal population, elevated ACE2 after chemotherapy is a predictor of poor response to treatment.

**Table 1:** The level of ACE2 in plasma of breast cancer patients

Group	Breast cancer patients	ACE2 (ng/mL)	*P*-value
Chemo-resistant	57	17.46	< 0.0001
Chemo-sensitive	54	13.47	

### Chemotherapy-induced expression of ACE2 is specifically detected in breast cancer

**Fig. 3:**
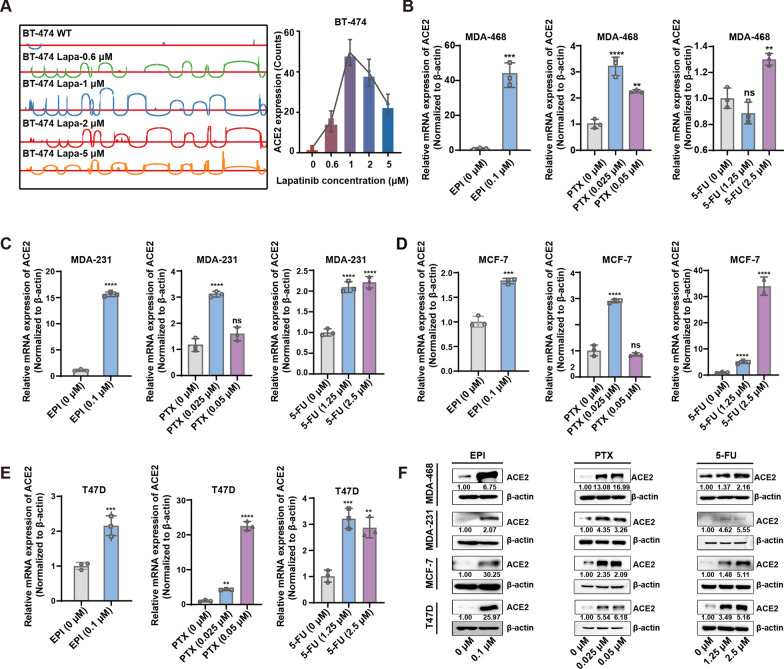
ACE2 is a chemotherapeutic drug-responsive gene specifically for breast cancer. **A** The increased reads of ACE2 during the acquisition of lapatinib resistance in BT-474 cells investigated by IGV. **B** qRT-PCR analysis showed elevated ACE2 expression in MDA-468, **C** MDA-231, **D** MCF-7 and **E** T47D cells exposed to low doses of EPI (0.1 µM), PTX (0.025 µM and 0.05 µM) and 5-FU (1.25 µM and 2.5 µM). **F** Western blot analysis showed ACE2 expression after exposure to EPI, PTX or 5-FU in MDA-468, MDA-231, MCF-7 and T47D cells. All data are shown as mean ± SD; **P < 0.01, ***P < 0.001, ****P < 0.0001, and ns *P* > 0.05 versus control

It is well known that the acquisition of resistance is a gradual process. To explore whether the upregulation of ACE2 occurs immediately when cells are exposed to anti-cancer drugs or after they acquire resistance. During the establishment of the lapatinib-resistant BT-474/Lapa cell model, we collected total RNA from cells resistant to different concentrations of the drug and performed transcriptome sequencing. As shown in Fig. 3A, the response of elevated ACE2 started at exposure to low concentrations of lapatinib (0.6 µM), peaked at increasing drug concentrations (1 µM), then partially subsided (2 µM) and maintained high-level of expression after acquisition of a stable resistant phenotype (5 µM). This data suggested that ACE2 expression is elevated when cancer cells are initially exposed to drugs and may play a role in acquiring a drug-resistant phenotype. Therefore, we hypothesized that ACE2 might be a chemotherapeutic drug-responsive gene. To prove our conjecture, we exposed breast cancer cells (MDA-468, MDA-231, MCF-7, T47D) with low doses of chemotherapeutic drugs for 72 h and examined ACE2 expression by qRT-PCR and western blot. The concentrations of these drugs were lower than the IC_50_ of cells collected from GDSC (Additional file 1: Table S5). As shown in Fig. 3B, the mRNA levels of ACE2 were significantly increased in EPI-exposed breast cancer cells (MDA-468) compared with the controls (Fig. 3B). Similarly, significant increases of ACE2 mRNA were also observed in MDA-468, MDA-231, MCF-7 and T47D cells exposed to PTX or 5-FU, which strongly supports our hypothesis (Fig. 3B–E). Consistently, chemotherapy-induced ACE2 expression was also detected at the protein level by Western blot assay (Fig. 3F). However, in colorectal and pancreatic cancer cells, the expression of ACE2 was not significantly increased after exposure to EPI, PTX or 5-FU (Additional file 2: Fig. S1). Taken together, these results demonstrate that ACE2 is a chemotherapeutic drug-responsive gene specifically for breast cancer.

### Chemotherapeutic drug-induced expression of ACE2 in breast cancer cells is ROS-dependent

**Fig. 4:**
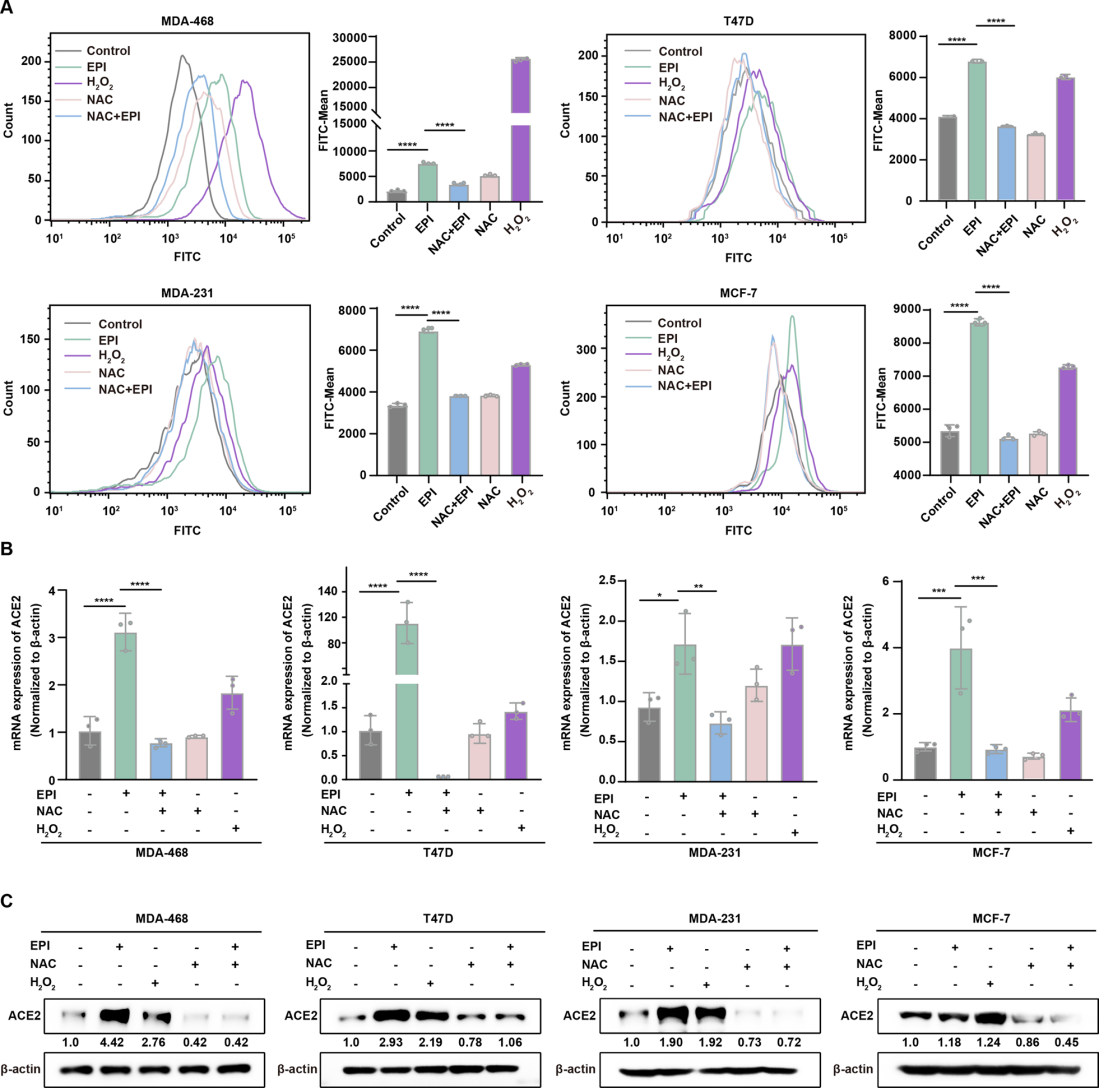
Chemotherapeutic drug-induced expression of ACE2 in breast cancer cells is ROS-dependent. **A** Low-dose chemotherapeutic drugs induce generation of ROS in breast cancer cells. MDA-468, T47D, MCF-7 and MDA-231 cells were treated with 0.05 µM EPI, 500 µM NAC, 0.05 µM EPI + 500 µM NAC at 37 °C for 72 h or 100 µM H_2_O_2_ for 4 h. Intracellular ROS levels were measured by flow cytometry using a Reactive Oxygen Species Assay Kit. The levels of ROS are summarized in the histogram on the right. **B** Western blot and **C** qRT-PCR analysis showed ACE2 expression in MDA-468, MDA-231, MCF-7 and T47D cells after exposure to EPI, NAC H_2_O_2_ or EPI + NAC. All data are shown as mean ± SD; *P < 0.05, **P < 0.01, ***P < 0.001, ****P < 0.0001, and ns *P* > 0.05 versus control

Considering that chemotherapeutic drugs induce oxidative stress in cancer cells, and the level of reactive oxygen species (ROS) is related to the expression of ACE2 [32, 42], we hypothesized that the expression of ACE2 induced by chemotherapeutic drugs might be related to ROS. Thus, ROS levels in EPI-treated breast cancer cells were evaluated using H_2_O_2_ (100 µM) as a positive control and the ROS scavenger NAC (500 µM) as a negative control. As shown in Fig. 4A, drug treatment significantly increased the level of ROS in breast cancer cells, while ROS levels increased after the addition of H_2_O_2_ and decreased after NAC treatment. To identify the dependence of drug-induced ACE2 elevation on ROS, breast cancer cells were treated with EPI in the presence or absence of the ROS scavenger NAC for 72 h. The results showed that the drug-induced increase in ROS levels was inhibited by the addition of NAC (Fig. 4A). Moreover, the expression of ACE2 in EPI-treated breast cancer cells was measured by qRT-PCR and western blotting in the presence or absence of NAC (Fig. 4B, C). The results showed that the EPI-induced increase in ACE2 in breast cancer cells was significantly attenuated by the ROS scavenger NAC. Together, these data suggest that chemotherapeutic drug-induced expression of ACE2 in breast cancer cells is dependent on its upregulation of intracellular ROS levels.

### HIF-1α is directly involved in the regulation of ACE2 expression in breast cancer cells by drug-induced ROS

**Fig. 5:**
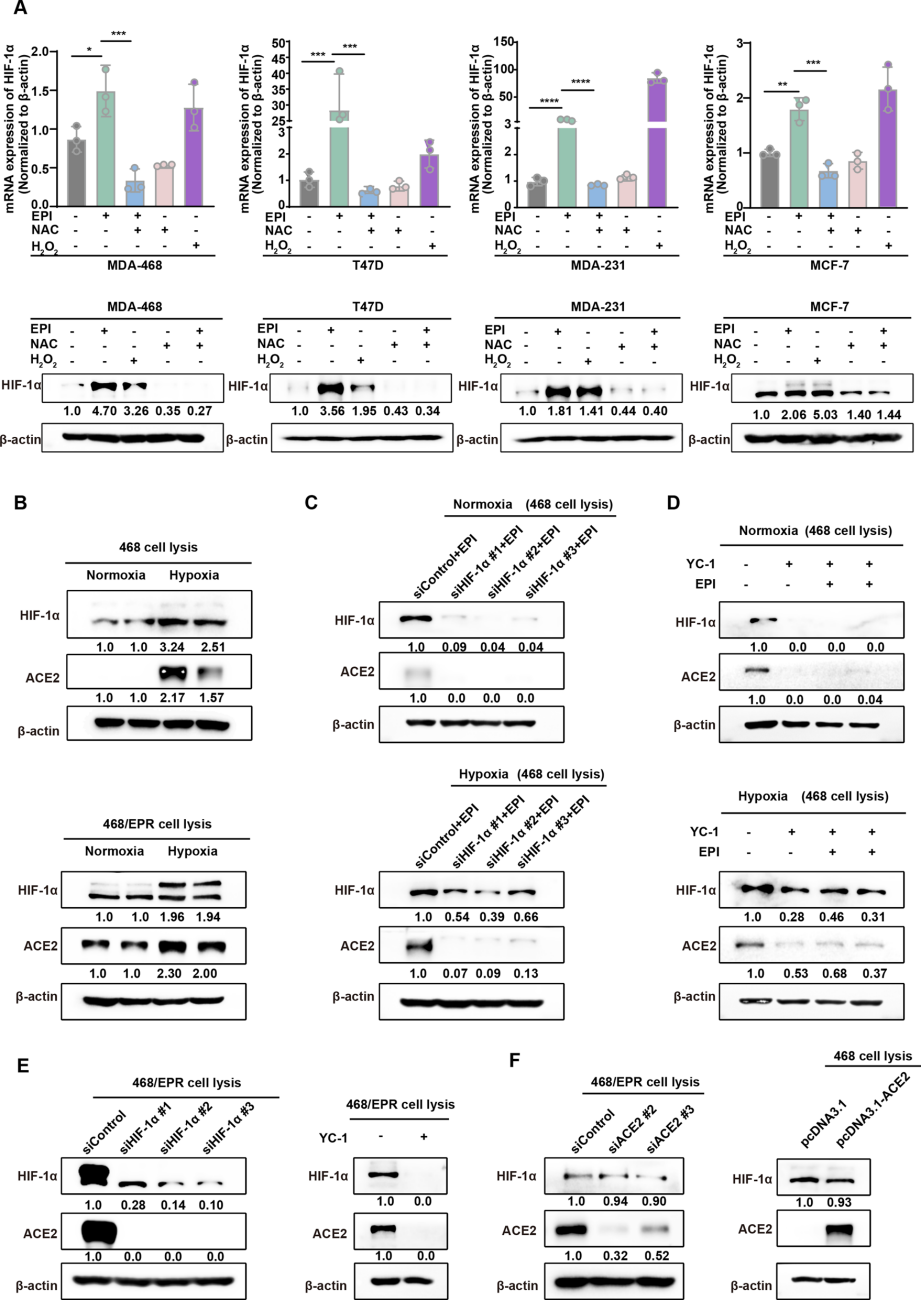
HIF-1α is directly involved in the regulation of chemotherapeutic drug-induced, ROS-mediated ACE2 expression. **A** Western blot and qRT-PCR analysis showed HIF-1α expression in four breast cancer cells after exposure to 0.05 µM EPI, 500 µM NAC, 0.05 µM EPI + 500 µM NAC for 72 h or 100 µM H_2_O_2_ for 4 h. **B** Western blotting analysis of ACE2 and HIF-1α expression in parental and resistant breast cancer cells cultured under normoxic or hypoxic (1% O_2_ for 24 h) conditions. The relative expression of proteins is quantified by grayscale analysis and is shown below the bands. **C** Knockdown of HIF-1α in MDA-468 cells or **D** inhibition of HIF-1α by YC-1 inhibitor blocked the EPI-induced elevation of ACE2 in breast cancer cells under normoxia or hypoxia. The relative expression of proteins is quantified by grayscale analysis and is shown below the bands. **E** Western blot analysis showed that both knockdown and inhibition of HIF-1α expression significantly decreased ACE2 expression in drug-resistant breast cancer cells (468/EPR cells). β-actin was used as an internal reference protein. **F** Western blot analysis showed no change in the protein level of HIF-1α after knockdown of ACE2 in 468/EPR cells and overexpression of ACE2 in MDA-468 cells. β-actin was used as a loading control

Recently, some evidence suggests an association between HIF-1α and ACE2 expression [31, 43]. ROS is known to regulate HIF-1α stabilization [44]. This raises a possibility that the increase in ACE2 expression induced by anticancer agents may involve ROS-mediated HIF-1α expression. Hence, we first analyzed the relationship between increased ROS levels by chemotherapeutic drugs and HIF-1α expression in breast cancer cells. Figure 5A showed that the mRNA and protein expression levels of HIF-1α were upregulated in four breast cancer cells after EPI treatment, while quenching of ROS with NAC counteracted the EPI-induced elevation of HIF-1α expression. Thus, we hypothesized that HIF-1α promotes EPI-induced ACE2 expression. It is known that HIF-1α protein is stable under hypoxic conditions. We then cultured breast cancer cells under normoxic and hypoxic conditions, respectively. As expected, the abundance of HIF-1α protein was significantly increased in breast cancer cells under hypoxic conditions, and the expression of ACE2 was also significantly upregulated. To further confirm the role of HIF-1α in regulating ACE2 expression, we knocked down HIF-1α expression using small interfering RNA (siRNA) in 468 cells, and then treated the cells with EPI for 72 h. As shown in Fig. 5C, EPI-induced upregulation of ACE2 was suppressed by siHIF-1α under either normoxic or hypoxic conditions. Consistently, the HIF-1α inhibitor YC-1 also blocked chemotherapy-induced expression of ACE2 (Fig. 5D). These results further demonstrate that HIF-1α is directly involved in the regulation of chemotherapeutics-induced ACE2 expression. We next investigated the effect of HIF-1α on ACE2 expression in drug-resistant breast cancer cells (468/EPR cells). Knockdown of HIF-1α expression with siRNA resulted in a decrease in ACE2 protein level in 468/EPR cells (Fig. 5E). Consistently, treatment of 468/EPR cells with YC-1 also reduced the expression of ACE2 (Fig. 5E). Nevertheless, the protein level of HIF-1α did not appear to be altered by overexpression of ACE2 in parental 468 cells and knockdown of ACE2 in resistant 468/EPR cells (Fig. 5F). Hence, HIF-1α acts upstream of ACE2 to regulate its expression. Collectively, these data suggest that HIF-1α is required for chemotherapeutic agent-induced, ROS-mediated ACE2 expression in breast cancer cells.

### ROS-dependent AKT activation mediates chemotherapeutic drug-induced ACE2 expression in breast cancer cells through regulation of HIF-1α

**Fig. 6:**
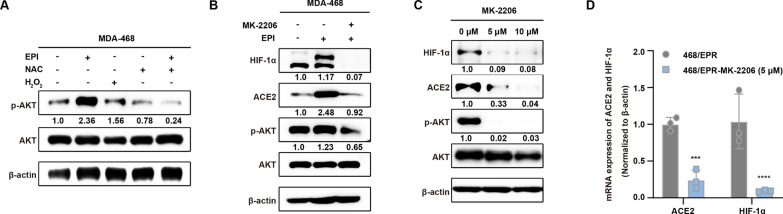
ROS-dependent AKT activation mediates chemotherapeutic drug-induced elevation of ACE2 in breast cancer cells through regulation of HIF-1α. **A** Western blot analysis of the expression of AKT and p-AKT in MDA-468 cells treated with EPI, NAC, H_2_O_2_ or EPI + NAC. **B** Inhibition of AKT phosphorylation by MK2206 completely blocked the expression of ACE2 and HIF-1α in EPI-induced breast cancer cells. **C** Western blot and **D** qRT-PCR showed that the expression levels of HIF-1α and ACE2 were downregulated in resistant breast cancer cells after inhibition of AKT phosphorylation. The numbers below the bands refer to the intensity of the relative grayscale values. β-actin was also used as the internal reference for qRT-PCR analysis

We next sought to identify the cellular mechanism by which chemotherapeutic drugs regulate ACE2 expression via HIF-1α. Several studies have suggested that high levels of ROS act as signaling molecules to affect the activation of intracellular pathways, such as promoting the phosphorylation of AKT [45]. Therefore, the relationship between AKT activation and ROS levels was assayed by Western blotting. As expected, increased levels of phospho-AKT were detected after EPI treatment, whereas the addition of the ROS scavenger NAC counteracted the EPI-induced increase in AKT phosphorylation (Fig. 6A). To determine whether AKT activity is required for chemotherapeutic drug-induced expression of HIF-1α and ACE2, we first treated parental breast cancer cells with the AKT inhibitor MK-2206 to determine the expression of AKT and HIF-1α and ACE2. As shown in Fig. 6B, MK-2206 significantly suppressed EPI-induced AKT phosphorylation and completely inhibited EPI-induced expression of HIF-1α and ACE2. Then, 468/EPR cells were exposed to MK-2206. As shown in Fig. 6C and D, inhibition of AKT phosphorylation reduced the expression of HIF-1α and ACE2 at the protein and mRNA levels. Taken together, these results suggest that EPI-induced expression of HIF-1α and ACE2 in breast cancer cells is highly dependent on ROS-mediated AKT activation.

### Knockdown of ACE2 reverses resistance to Epirubicin but promotes proliferation of drug-resistant breast cancer cells

**Fig. 7:**
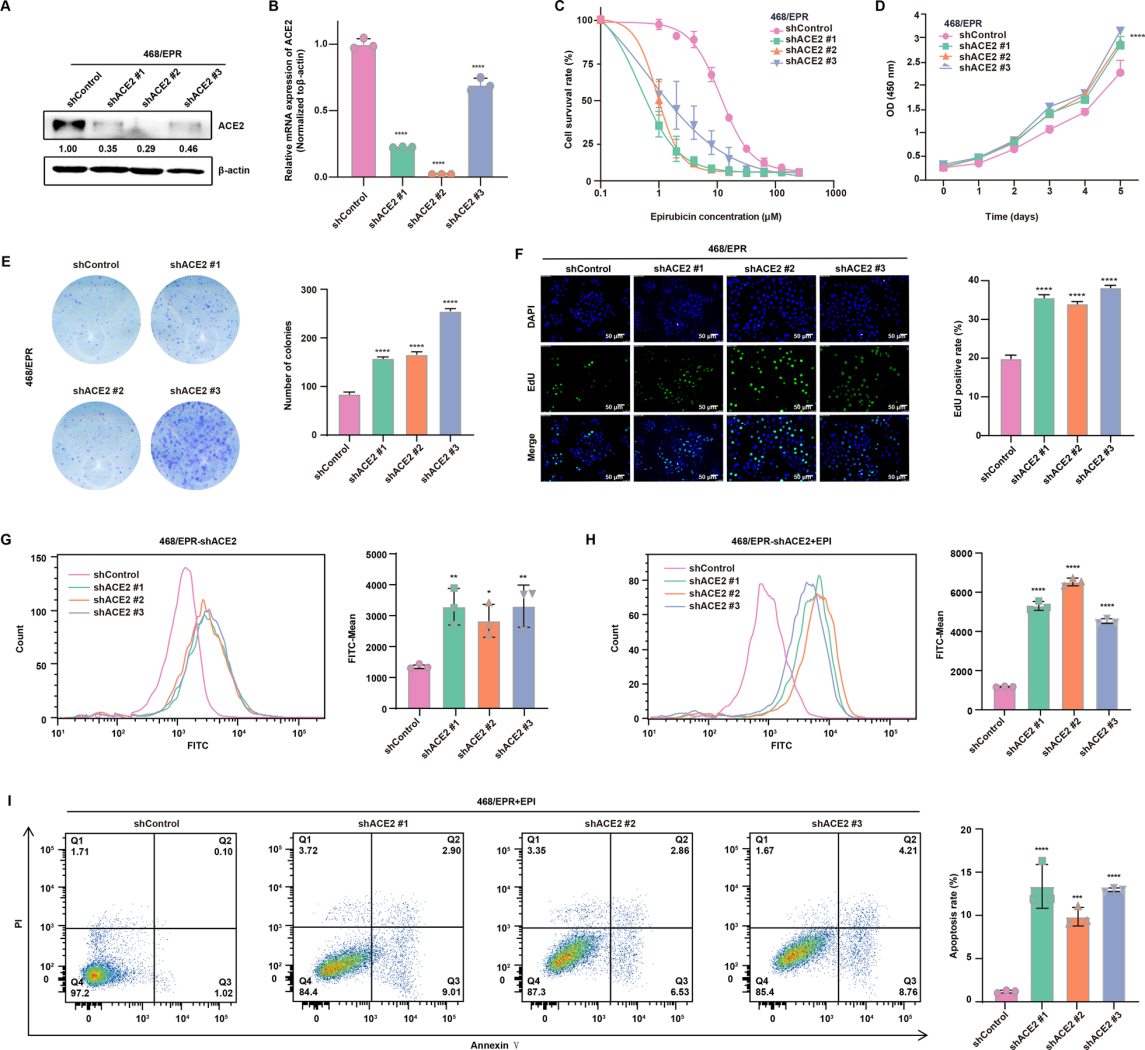
Knockdown of ACE2 reverses resistance to Epirubicin but promotes the proliferation of drug-resistant breast cancer cells. **A** Western blot and **B** qRT-PCR assays verified that ACE2 was successfully knocked down by three independent shRNA targeting ACE2 in 468/EPR cells. **C** Silencing ACE2 in 468/EPR cells significantly increased the sensitivity to EPI compared with shControl and wild-type cells. The IC_50_ assay was performed by using the CCK-8 based method. **D** Silencing ACE2 in 468/EPR cells significantly increased cell proliferative activity compared with shControl cells (****P < 0.0001). **E** Knockdown of ACE2 in 468/EPR cells remarkably increased the formation of cell colonies compared with shControl cells (****P < 0.0001). **F** EdU cell proliferation assay displayed that knockdown of ACE2 expression resulted in a significant increase in EdU positive rate, indicating higher cell proliferation ability. **G** Intracellular ROS levels were remarkably increased in ACE2 knockdown 468/EPR cells than in shControl cells. **H** Intracellular ROS levels were increased approximately 4-fold in 468/EPR-shACE2 cells treated with 0.05 µM EPI for 72 h compared to control cells. **I** Knockdown of ACE2 significantly increased the rate of EPI-induced apoptosis in 468/EPR cells compared with the control group. All data are shown as mean ± SD; *P < 0.05, *P < 0.01, ***P < 0.001, ****P < 0.0001, and ns *P* > 0.05 versus control

To determine the functional role of ACE2 in drug-resistant breast cancer cells, we next stably silenced the expression of ACE2 in 468/EPR cells using lentivirus expressing ACE2-specific shRNAs. As shown in Fig. 7A and B, Western blot and qRT-PCR analysis showed that ACE2 expression was substantially reduced in ACE2 shRNA-infected cells compared with that of control shRNA-infected cells. Then, the sensitivity of ACE2-silenced cells to EPI was investigated. As shown in Fig. 7C and Table 2, the survival rate of resistant cells exposed to EPI was decreased after ACE2 knockdown, and the IC_50_ value of EPI was significantly reduced in ACE2-silenced cells compared with control cells. By contrast, CCK8-based assay showed that ACE2 silencing significantly increased the proliferative activity of resistant cells (Fig. 7D). Similarly, 468/EPR-shACE2 cells also showed remarkably increased colony-forming capacity in vitro compared with control cells (Fig. 7E). EdU staining also confirmed that decreased expression of ACE2 significantly increased the cell proliferation index (Fig. 7F). Hence, knockdown of ACE2 in resistant cancer cells resulted in increased proliferative capacity compared to control cells. Since chemotherapeutic drug-induced ACE2 elevation was related to increased ROS levels, we therefore determined the changes in intracellular ROS levels in 468/EPR-shACE2 cells. Interestingly, knockdown of ACE2 resulted in an approximately 2-fold increase in intracellular ROS levels in 468/EPR cells compared to control cells (Fig. 7G). Moreover, ROS levels were increased approximately 4-fold in 468/EPR-shACE2 cells treated with 0.05 µM EPI for 72 h compared to control cells (Fig. 7H). Thus, knockdown of ACE2 resulted in increased intracellular ROS levels, and EPI induced a large increase in ROS levels in ACE2 knockdown cells. Furthermore, Annexin-V/PI staining assay displayed that the apoptosis rate of 468/EPR-shACE2 cells was significantly increased after 3 days of EPI treatment compared with the controls (Fig. 7I). Collectively, these results suggest that although ACE2 knockdown accelerates cell proliferation, it may lead to an excessive elevation of EPI-induced intracellular ROS, which in turn induces apoptosis and thus enhances cell sensitivity to chemotherapeutic agents.

**Table 2:** IC_50_ values in control and ACE2 knockdown 468/EPR cells

Cell	IC_50_	95% CI (profile likelihood)	*P*-value
468/EPR shControl	11.53	10.89 to 12.21	
468/EPR shACE2 #1	0.47	0.3717 to 0.5565	0.0316
468/EPR shACE2 #2	0.94	0.8993 to 0.9837	0.0347
468/EPR shACE2 #3	0.68	0.2337 to 1.085	0.0345

### ACE2 is strongly positively correlated with breast cancer drug resistance genes

**Fig. 8:**
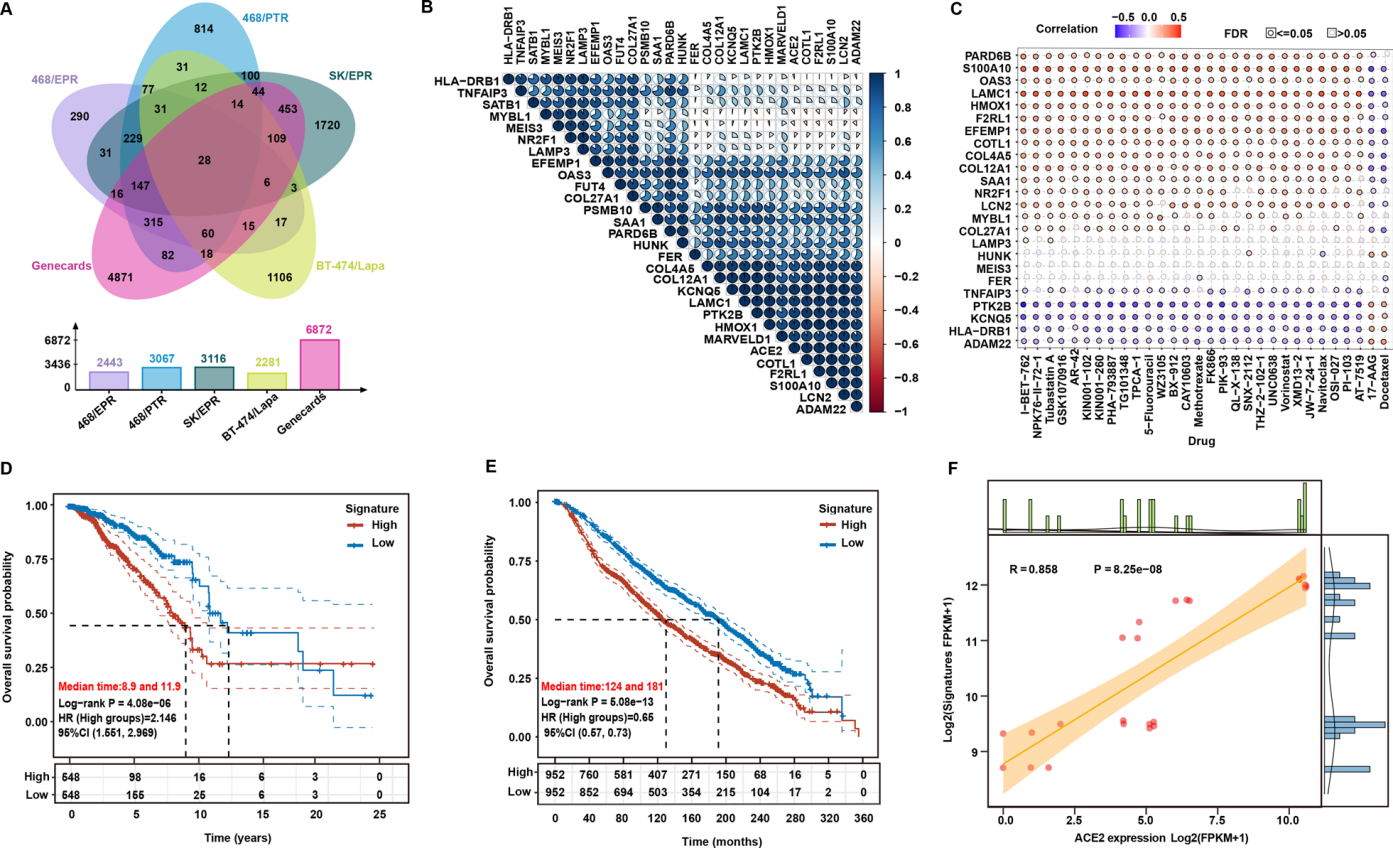
ACE2 is strongly positively correlated with breast cancer drug resistance genes (BRCA-DRGs). **A** The Venn diagram of common upregulated genes between four groups of drug-resistant cells and breast cancer drug-resistant genes defined by GeneCards. The barplot below Venn diagram shows the detailed number of each gene list. **B** Heatmap showing the correlation of genes expression in breast cancer drug resistance gene set. **C** The bubble plot summarizing the correlations between BRCA-DRGs and anticancer drugs. **D** Kaplan–Meier survival analysis of the signature (BRCA-DRGs) in TCGA-BRCA and (E) METABRIC databases. **F** Spearman correlation analysis of ACE2 expression and the signature (BRCA-DRGs). Scatter plot showed that the expression of ACE2 and the signature (BRCA-DRGs) was positively correlated in breast cancer drug-resistant cells (R = 0.858, *P* = 8.25e−08)

Although we have initially established a positive correlation between ACE2 elevation and chemotherapy resistance in breast cancer, whether ACE2 is involved in chemoresistance in breast cancer still requires further investigation. To determine the relationship between ACE2 and drug resistance, we first constructed a drug resistance gene set using bioinformatics analysis based on the above transcriptome sequencing data and public database. As shown in Fig. 8A, a 28 genes signature (named as BRCA-DRGs, Table 3) was identified by combined analysis of the upregulated genes (LogFC > 1 and FDR < 0.05) in the four groups of drug-resistant/parental RNA-Seq data and drug-resistant genes defined by GeneCards. Expectedly, a strong positive correlation pattern was observed among these genes in our RNA-sequencing results of the four drug-resistant/parental cells, indicating that these genes are closely interconnected in drug-resistant cells (Fig. 8B). We next validated the correlation between BRCA-DRGs and drug sensitivity of cancer cells in the GDSC database. As shown in Fig. 8C, most of these genes were positively correlated with increased resistance of cancer cells to anticancer drugs, indicating that our constructed BRCA-DRGs could well predict the sensitivity of cancer cells to drugs. Moreover, survival analysis based on TCGA-BRCA and METABRIC database further showed that patients with high expression of BRCA-DRGs had poorer overall survival time (Fig. 8D and E). Notably, we also identified a strong positive correlation between ACE2 and BRCA-DRGs (R = 0.858, *P* = 8.25e−08) (Fig. 8F), indicating that ACE2 may be involved in regulating drug resistance in breast cancer.

**Table 3:** The list of breast cancer drug-resistant genes set (BRCA-DRGs).

Gene name	Gene description	Chrom
FUT4	Fucosyltransferase 4	chr11
PARD6B	Par-6 family cell polarity regulator beta	chr20
HLA-DRB1	Major histocompatibility complex class II DR beta 1	chr6
HUNK	Hormonally up-regulated Neu-associated kinase	chr21
EFEMP1	EGF containing fibulin-like extracellular matrix protein 1	chr2
COL27A1	Collagen type XXVII alpha 1	chr9
NR2F1	Nuclear receptor subfamily 2 group F member 1	chr5
MEIS3	Meis homeobox 3	chr19
PSMB10	Proteasome subunit beta 10	chr16
COL4A5	Collagen type IV alpha 5	chrX
F2RL1	Coagulation factor II (thrombin) receptor-like 1	chr5
KCNQ5	Potassium channel voltage gated KQT-like subfamily Q member 5	chr6
TNFAIP3	TNF alpha induced protein 3	chr6
COL12A1	Collagen type XII alpha 1	chr6
PTK2B	Protein tyrosine kinase 2 beta	chr8
S100A10	S100 calcium binding protein A10	chr1
LCN2	Lipocalin 2	chr9
ADAM22	ADAM metallopeptidase domain 22	chr7
LAMP3	Lysosomal-associated membrane protein 3	chr3
MYBL1	v-myb avian myeloblastosis viral oncogene homolog-like 1	chr8
HMOX1	Heme oxygenase 1	chr22
FER	fer (fps/fes related) tyrosine kinase	chr5
SAA1	Serum amyloid A1	chr11
SATB1	SATB homeobox 1	chr3
OAS3	2′-5′-oligoadenylate synthetase 3	chr12
MARVELD1	MARVEL domain containing 1	chr10
LAMC1	Laminin subunit gamma 1	chr1
COTL1	Coactosin-like F-actin binding protein 1	chr16

### Elevated ACE2 expression is a marker of poor prognosis in breast cancer patients receiving chemotherapy

**Fig. 9:**
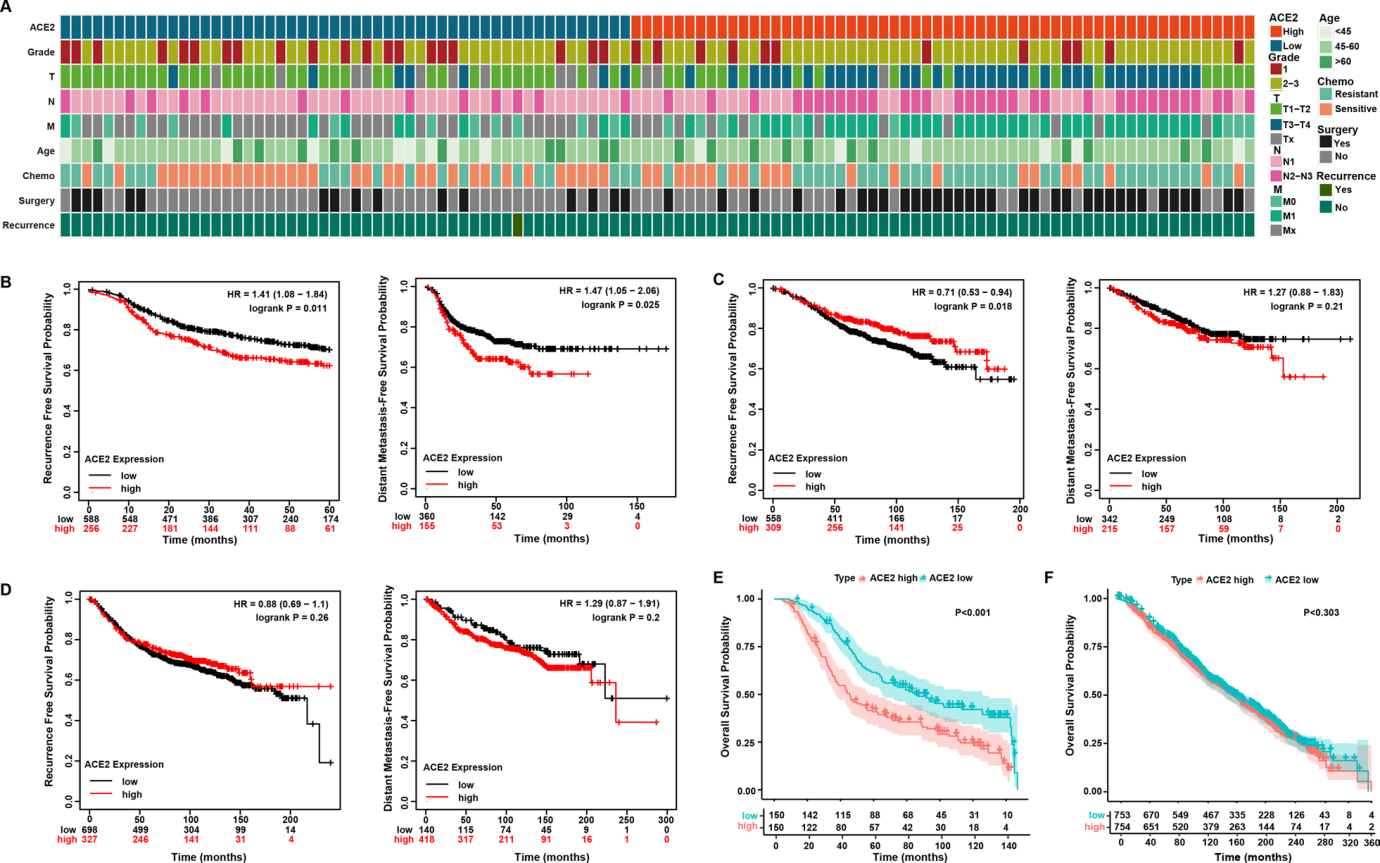
Elevated ACE2 expression is a marker of poor prognosis in breast cancer patients receiving chemotherapy. **A** Waffle Chart of clinicopathological characteristics of breast cancer patients with high and low ACE2 expression. **B** Kaplan–Meier curves of the RFS and DMFS in BRCA patients receiving chemotherapy with high or low ACE2 expression (KM Plotter database). **C** Kaplan–Meier curves of the RFS and DMFS in ACE2 high and low expression groups in BRCA patients who did not receive chemotherapy (KM Plotter database). **D** Kaplan–Meier curves of the RFS and DMFS of systematically untreated BRCA with high or low ACE2 expression (KM Plotter database). **E** Kaplan–Meier survival analysis of OS in ACE2 high and low expression groups in the METABRIC cohort of breast cancer patients receiving chemotherapy and **F** breast cancer patients not receiving chemotherapy

To investigate the relationship between ACE2 expression in plasma and clinical prognostic parameters of breast cancer, we chose the median value of ACE2 expression as the cutoff value and divided the samples into ACE2 high and low groups (Table 4). Waffle Chart was generated based on prognostic parameters and ACE2 expression (Fig. 9A). The results showed that higher ACE2 expression was positively correlated with poor differentiation (*P =* 0.031, Table 4), large tumor volume (*P =* 0.004, Table 4) and poor response to chemotherapy (*P =* 0.006 Table 4). Due to the short follow-up time of the patients whose plasma was collected in this study, the effect of ACE2 expression on breast cancer prognosis could not be analyzed. Therefore, we analyzed the prognostic impact of ACE2 expression by Kaplan-Meier plotter from public databases including GEO, EGA, and TCGA. As shown in Fig. 9B, among breast cancer patients receiving chemotherapy, both recurrence-free survival (RFS) and distant metastasis-free survival (DMFS) were significantly lower in patients with high ACE2 expression than in those with low expression. However, there was no significant association between ACE2 expression and survival in breast cancer patients who did not receive chemotherapy and were systematically untreated (Fig. 9C, D). Furthermore, we verified the same results in another database, METABRIC. As shown in Fig. 9E, in breast cancer patients receiving chemotherapy, high ACE2 expression was associated with poorer overall survival, whereas ACE2 expression was not significantly associated with overall survival in patients who did not receive chemotherapy. Altogether, these results suggest that elevated ACE2 expression is a marker of poor prognosis in breast cancer patients receiving chemotherapy.

**Table 4:** Clinicopathological characteristics of breast cancer patients with high and low ACE2 expression

Variables	ACE2		*P*-value
	Higher (> 14.78)	Lower (< 14.78)	
	N = 56	N = 55	
Mean-Age (years)	52.89	50.2	
ACE2 (ng/mL)	21.23	9.71	
Chemotherapy			
Chemotherapy-resistant	36(64.29%)	21(38.18%)	0.0059
Chemotherapy-sensitive	20(35.71%)	34(61.81%)	
Grade			
I	11(19.64%)	21(38.18%)	0.0311
II–III	45(80.36%)	34(61.82%)	
T			
T1	9(16.07%)	20(36.36%)	0.0040
T2–T3	41(73.21%)	24(43.64%)	
Tx	2(3.57%)	11(20.00%)	
N			
N0	19(33.93%)	28(3.63%)	0.0700
N1–N2–N3	37(66.07%)	27(96.37%)	
M			
M0	10(17.86%)	11(20.00%)	0.0077
M1	36(64.29%)	9(16.36%)	
MX	10(17.86%)	35(63.63%)	
Surgery			
Yes	33 (58.93%)	15 (27.27%)	0.0008
No	23 (41.07%)	40 (72.72%)	
Recurrence			
Yes	0 (0%)	1 (1.82%)	0.3108
No	56 (100%)	54 (98.18%)	
Metastasis			
Yes	6 (10.71%)	23 (41.82%)	0.0002
No	50 (89.29%)	32 (58.18%)	

## Discussion

**Fig. 10:**
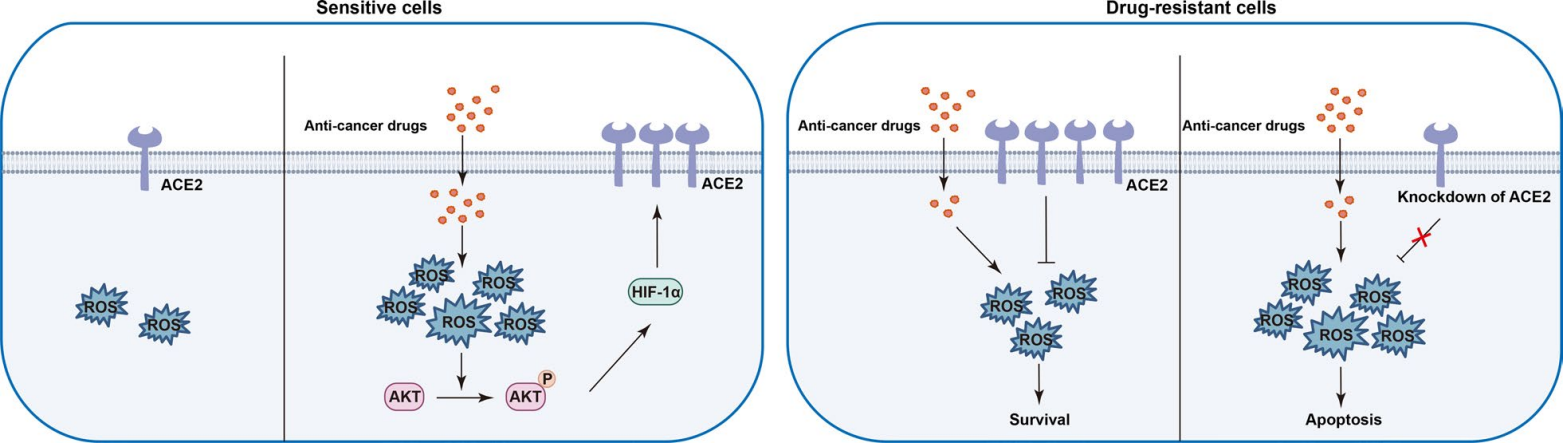
Schematic illustration of the relationship between ROS levels and ACE2 during chemotherapy in breast cancer cells. Left panel: In sensitive breast cancer cells with low ROS levels, ACE2 expression is relatively low; when the cells are stimulated by chemotherapeutic agents, ROS production is induced and phosphorylation of AKT is promoted, which in turn upregulates ACE2 expression by enhancing the expression of HIF-1α. Right panel: ACE2 is expressed at high levels in drug-resistant cells and maintains ROS homeostasis by inhibiting the overproduction of ROS. In drug-resistant cells with knockdown of ACE2, anticancer drugs induced a large increase in ROS levels, which induced apoptosis

The most significant findings of this study provide novel insights into the role of ACE2 in the acquisition of drug resistance and progression of breast cancer. As illustrated in Fig. 10, we demonstrate that ACE2 expression is relatively low in breast cancer cells, but its expression increases specifically and rapidly after exposure to anticancer drugs, and stabilizes at a high level after acquisition of drug resistance. Mechanistically, chemotherapeutic agents induce ACE2 expression in breast cancer cells by increasing intracellular ROS production, while increased ROS levels enhance AKT phosphorylation and subsequently upregulate HIF-1α expression. Although ACE2 levels in plasma and cancer tissues are significantly lower in breast cancer patients compared with healthy controls, elevated ACE2 in patients after chemotherapy is a predictor of poor response to treatment. Importantly, patients with high ACE2 expression had significantly lower RFS and DMFS compared with patients with low expression. Moreover, ACE2 may be involved in regulating drug resistance and proliferation of breast cancer cells by optimizing the balance of intracellular ROS. Collectively, our results highlight the key functional role of ACE2 in breast cancer progression and treatment.

Aberrant expression of ACE2 is observed in several cancers and correlates with patient prognosis. In most cases, ACE2 expression is lower in tumor tissues than in normal tissues, and patients with high ACE2 levels in cancer tissues appear to be associated with a favorable prognosis [27, 28, 46]. Nevertheless, other studies have reported opposite results that ACE2 upregulation promotes tumor progression, and even that ACE2 may play distinct roles in different subtypes of the same tumor [47, 48]. This feature seems to be more pronounced in breast cancer [24, 49, 50]. Despite previous studies have reported the relationship between ACE2 expression and breast cancer prognosis, a systemic investigation of the association between ACE2 expression and chemotherapy and prognosis is still lacking. Herein, we found reduced ACE2 expression in plasma of breast cancer patients compared with healthy controls, which is similar to the results of our analysis in the TCGA database. These data are consistent with previous studies showing that ACE2 is higher in normal tissues than in cancer tissues. Hence, ACE2 may act a tumor suppressor during cancer initiation. Interestingly, patients with high plasma ACE2 were less sensitive to chemotherapy. Similarly, analysis from the METABRIC database also revealed an inverse relationship between ACE2 expression and response to chemotherapy in breast cancer patients. Additionally, ACE2 was also specifically upregulated in drug-resistant breast cancer cells. Thus, our study identified a novel link between ACE2 and breast cancer, whereby elevated ACE2 in tumors is associated with treatment resistance in patients. These findings also indicate temporal and spatial differences in the role of ACE2 in tumorigenesis and progression. To our knowledge, this is the first report on ACE2 expression and treatment resistance in breast cancer.

Our findings suggest that ACE2 is a specific drug response gene in breast cancer. Anti-cancer agents are known to increase ROS and cause oxidative stress in cancer cells, and ACE2 is able to maintain cell viability by reducing oxidative stress in certain cell types [51, 52]. Therefore, elevated ACE2 is likely induced by drug-caused oxidative stress and has a protective effect on cancer cells after chemotherapy. Consistently, ACE2 expression persisted until the cells acquired a drug-resistant phenotype, suggesting that elevated ACE2 may promote cell survival by reducing ROS and maintaining intracellular redox homeostasis, as excessive production of ROS may lead to oxidative stress and cell death. Subsequent studies confirmed this possibility, with intracellular ROS levels significantly increased in ACE2-silenced cells and anticancer drugs inducing higher levels of ROS production in ACE2-knockdown cells. These findings also raised a possibility that ACE2 upregulation in breast cancer cells may modulate the sensitivity of cells to drugs. As expected, silencing ACE2 significantly reversed the resistance of breast cancer cells to chemotherapeutic drugs. Likewise, ACE2-silenced cells showed enhanced apoptosis rate after EPI treatment. Interestingly, cell proliferation was enhanced after knockdown of ACE2 in drug-resistant cells under normal culture conditions. We speculate that this may be partly due to the elevated intracellular ROS levels caused by ACE2 knockdown, as moderate ROS facilitates cell survival and proliferation. Nevertheless, ACE2 silencing deprives cells of the ability to reduce the excess ROS induced by anticancer drugs, thereby increasing the rate of apoptosis and decreasing drug resistance. Consistently, ACE2 expression was strongly correlated with drug-resistant genes signature (BRCA-DRGs) constructed in our study, further supporting the involvement of ACE2 in the regulation of drug resistance in breast cancer. Consequently, ACE2 promotes drug resistance by regulating intracellular ROS-induced oxidative stress. Notably, oxidative stress-induced ACE2 upregulation was also seen in other cell types and stimuli [53]. Smoking is known to induce oxidative stress and ROS production in respiratory cells [54]. A recent study showed that smoking can lead to ACE2 overexpression in bronchial and alveolar epithelial cells [31]. In addition, inflammation-related stress can also contribute to ACE2 upregulation [55]. Several consistent findings suggest that certain inflammatory cytokines also trigger upregulation of ACE2 in different models [32]. Thus, ACE2 expression tends to be elevated when cells are subjected to noxious stimuli, suggesting a functional response of ACE2 to external stimuli. Collectively, these findings suggest that anticancer drugs induce ACE2 expression by increasing intracellular ROS, which in turn reduces excessive elevation of ROS, thereby maintaining the balance of intracellular oxidative stress, sustaining cell survival and promoting drug resistance.

The molecular mechanism of ACE2 upregulation in response to chemotherapeutic drug is worth investigating. Here, we demonstrated that drug-induced elevation of ACE2 in breast cancer cells is ROS-dependent. ROS is known to play a double-edged role in cell viability [42]. Moderate ROS can promote cell survival and proliferation by activating a number of intracellular signaling pathways [56]. Excessive ROS levels can cause cell damage and death [57]. Our current results suggest that a low-dose drug-induced rise in ROS is a key factor contributing to elevated ACE2 expression. This scenario is most likely to exist in cancer patients during chemotherapy, as the anti-cancer drugs are not evenly distributed within the tumor, then cancer cells exposed to lower concentrations of drugs may upregulate ACE2 through this mechanism, and the elevated ACE2 allows the cells to survive by reducing oxidative stress damage caused by ROS. We further confirmed that this drug-induced, ROS-dependent upregulation of ACE2 was associated with the elevation of HIF-1α. Similarly, a recent study showed that HIF-1α could bind to the promoter region of ACE2 in respiratory cells and promote its transcription in response to smoking stimulation [31]. Although several reports have shown that HIF-1α negatively regulates the expression of ACE2 in some cells [58], this may be due to the differences in cell type or context as well as induction factors. In addition, HIF-1α is well-known to be regulated by the AKT pathway, and ROS as a signaling molecule can induce the activation of AKT [45, 59]. Consistently, EPI-induced elevation of AKT phosphorylation in breast cancer cells was counteracted by the ROS scavenger NAC. Meanwhile, AKT inhibition by MK-2206 reduced the expression of HIF-1α and ACE2 in both parental and resistant breast cancer cells. Collectively, our results suggest that EPI-induced ACE2 expression in breast cancer cells is dependent on the ROS-AKT-HIF-1α signaling pathway.

Another important clinical implication of this study is that elevated ACE2 expression is also a marker of poor prognosis in breast cancer patients receiving chemotherapy. Although previous reports have analyzed the relationship between ACE2 expression and the prognosis of breast cancer patients, few studies have focused on the association between its expression and clinical treatment and prognosis. Herein, we found that ACE2 expression did not appear to be significantly associated with overall prognosis in breast cancer patients who did not receive chemotherapy or systematically untreated. Nevertheless, in patients receiving chemotherapy, elevated ACE2 was correlated with worse RFS and DMFS compared with patients with low ACE2 expression. This finding is inconsistent with the result reported in a recent publication showing that elevated ACE2 immunostaining is unrelated to the outcome in nonspecific types of invasive breast cancer [49]. This inconsistency may be due to the fact that the aforementioned study only focused on the expression level of ACE2, but did not include the treatment modality of the patients. Our findings also imply that upregulation of ACE2 in cancer tissues with originally low ACE2 expression may be a driver of cancer progression. This possibility is partly supported by a recent study showing that higher ACE2 expression appears to be associated with higher histological grade, HER2-enriched and Basal-like subtypes [49]. These indicators are known to be associated with poor prognosis in breast cancer. Collectively, these results indicate spatiotemporal differences in the function of ACE2 in cancer initiation and progression.

In summary, our study demonstrated that ACE2 is a gene that responds rapidly to chemotherapeutic drugs via the ROS-AKT-HIF-1α axis, and specifically in breast cancer. The function of upregulated ACE2 in breast cancer cells may differ from normal cells. Elevated ACE2 modulates the sensitivity of breast cancer cells to anticancer regents. Moreover, elevated ACE2 was not only a predictor of poor response to chemotherapy, but is also correlated with worsen outcomes in breast cancer patients. In addition, it is worth noting that due to the COVID-19 pandemic, our study also suggests that breast cancer patients may exhibit higher ACE2 expression after chemotherapy, which increases the risk of infection with SARS-CoV-2 and contributes to high COVID-19 mortality in cancer patients [60].

## Supplementary Information

**Additional file 1: Table S1.** The primers used for qRT-PCR in this study. **Table S2.** The sequence of siRNAs target ACE2 and HIF-1α used in this study. **Table S3.** The sequence for shACE2 used in this study. **Table S4.** Clinicopathological characteristics of healthy donors and breast cancer patients enrolled in this study. **Table S5.** IC_50_ of breast cancer, colorectal cancer and pancreatic cancer cell lines in GDSC.

**Additional file 2: Figure S1.** (A) qRT-PCR analysis showed no significant changes in ACE2 expression in colorectal and pancreatic cancer cells after exposure to EPI, PTX or 5-FU. All data are shown as mean ± SD; *P < 0.05, **P < 0.01, ***P < 0.001, ****P < 0.0001, and ns *P* > 0.05 versus control, N = 3.

## Data Availability

The datasets used in this research are available in the following public databases: TCGA-BRCA (http://portal.gdc.cancer.gov/), METABRIC (http://www.cbioportal.org/), Gene Expression Omnibus (https://www.ncbi.nlm.nih.gov/gds) repository (GSE16080 and GSE54326), Cancer Cell Line Encyclopedia (CCLE) (https://portals.broadinstitute.org/ccle), and Genecards database (https://www.genecards.org/). In addition to datasets from public databases, other datasets used and analyzed during the present study are available from the corresponding author on reasonable request.

## Abbreviations

ACE2: Angiotensin-converting enzyme 2
SARS-CoV-2: Severe acute respiratory syndrome coronavirus 2
COVID-19: Coronavirus disease 2019
RAS: Renin-angiotensin system
Ang-II: Angiotensin-II
BRCA-DRGs: Breast cancer drug-resistant genes
IGV: Integrative Genomics Viewer
EPI: Epirubicin
PTX: Paclitaxel
5-FU: 5-Fluorouracil
RFS: Recurrence-free survival
DMFS: Distant metastasis-free survival
