# Supplementary material for: Chemotherapy induces ACE2 expression in breast cancer via the ROS-AKT-HIF-1α signaling pathway: a potential prognostic marker for breast cancer patients receiving chemotherapy
Source: J Transl Med. 2022 Nov 5;20:509. doi: 10.1186/s12967-022-03716-w (PMC9636712; doi:10.1186/s12967-022-03716-w)
Supplement: Supplementary file 1 — Additional file 1: Table S1. The primers used for qRT-PCR in this study. Table S2. The sequence of siRNAs target ACE2 and HIF-1α used in this study. Table S3. The sequence for shACE2 used in this study. Table S4. Clinicopathological characteristics of healthy donors and breast cancer patients enrolled in this study. Table S5. IC50 of breast cancer, colorectal cancer and pancreatic cancer cell lines in GDSC. [file 12967_2022_3716_MOESM1_ESM.docx]

Supplementary files for

**Chemotherapy induces elevated ACE2 in breast cancer via the ROS-AKT-HIF-1α signaling pathway: a potential prognostic marker for breast cancer patients receiving chemotherapy**

Xiaoyan Zuo^1,2,3,4^, Sixin Ren^1,2,3,4^, He Zhang^1,2,3,4^, Jianfei Tian^1,2,3,4^, Ruinan Tian^1,2,3,4^, Baoai Han^1,2,3,4^, Hui Liu^1,2,3,4^, Qian Dong^1,2,3,4^, Zhiyong Wang^1,2,3,4^, Yanfen Cui^1,2,3,4^, Ruifang Niu*, Fei Zhang*

^1^Public Laboratory, Tianjin Medical University Cancer Institute and Hospital, National Clinical Research Center for Cancer, Tianjin 300060, China.

^2^Key Laboratory of Cancer Prevention and Therapy, Tianjin 300060, China.

^3^Tianjin’s Clinical Research Center for Cancer, Tianjin 300060, China.

^4^Key Laboratory of Breast Cancer Prevention and Therapy, Tianjin Medical University, Ministry of Education, Tianjin 300060, China.

# Correspondence: Ruifang Niu, Fei Zhang; email: [rniu@tmu.edu.cn](mailto:rniu@tmu.edu.cn), [feizhang03@tmu.edu.cn](mailto:feizhang03@tmu.edu.cn)

**This PDF file includes**

Tables S1 to S5.

**The Supplementary Material for this manuscript includes the following:**

Supplementary files

**Tables**

**Table S1. The primers used for qRT-PCR in this study.**

| **gene** | | **Homo sapiens** | | |
| --- | --- | --- | --- | --- |
|  |  | **primer 5'-3'** | **Product length (bp)** | **OD** |
| ACE2 | GTGCACAAAGGTGACAATGG | | 160 | 2 |
|  | GGCTGCAGAAAGTGACATGA | |  | 2 |
| HIF-1α | ATCCATGTGACCATGAGGAAATG  TCGGCTAGTTAGGGTACACTTC | | 125 | 4 |
| β-actin | CTGGGTGTTGAAGGTCTC | | 216 | 4 |
|  | CAGAGCAAGAGAGGCATCC | |  | 4 |

**Table S2. The sequence of siRNAs target ACE2 and HIF-1α used in this study.**

| **Gene primer name** | **Squence (5'–3')** | |
| --- | --- | --- |
| siHIF-1α #1 | Forward | GCCGCUCAAUUUAUGAAUATT |
|  | Reverse | UAUUCAUAAAUUGAGCGGCTT |
| siHIF-1α #2 | Forward | CCACCACUGAUGAAUUAAATT |
|  | Reverse | UUUAAUUCAUCAGUGGUGGTT |
| siHIF-1α #3 | Forward | GCUGGAGACACAAUCAUAUTT |
|  | Reverse | AUAUGAUUGUGUCUCCAGCTT |
| siACE2 #1 | Forward | GCGAGUGGCUAAUUUGAAATT |
|  | Reverse | UUUCAAAUUAGCCACUCGCTT |
| siACE2 #2 | Forward | GGACAAGUUUAACCACGAATT |
|  | Reverse | UUCGUGGUUAAACUUGUCCTT |

**Table S3. The sequence for shACE2 used in this study.**

| **Gene primer name** | **Squence (5'–3')** | |
| --- | --- | --- |
| 468/EPR shACE2 #1 | Forward | GCAGCTGAGGCCATTATATGA |
|  | Reverse | TCATATAATGGCCTCAGCTGC |
| 468/EPR shACE2 #2 | Forward | GCTCATTTGCTTGGTGATATG |
|  | Reverse | CATATCACCAAGCAAATGAGC |
| 468/EPR shACE2 #3 | Forward | GCAGCCACACCTAAGCATTTA |
|  | Reverse | TAAATGCTTAGGTGTGGCTGC |

**Table S4. Clinicopathological characteristics of healthy donors and breast cancer patients enrolled in this study.**

| **Variables** | **Healthy Group (N=20)** | **Breast Cancer Patients (N=111)** | | |
| --- | --- | --- | --- | --- |
|  |  | **Total 111** | **Chemotherapy-resistant** | **Chemotherapy-sensitive** |
|  |  |  | 57 (51.35%) | 54 (48.65%) |
| **Age-year** |  |  |  |  |
| Mean | 44.35 | 51.55 | 52.14 | 50.94 |
| Range | 19-83 | 31-74 | 34-74 | 31-71 |
| **Grade** |  |  |  |  |
| Ⅰ | / | 32 (28.83%) | 7 (12.28%) | 25 (46.30%) |
| Ⅱ-Ⅲ | / | 79 (71.17%) | 50 (87.72%) | 29 (53.70%) |
| **T** |  |  |  |  |
| T1-T2 | / | 56 (50.45%) | 29 (50.88%) | 27 (50%) |
| T3-T4 | / | 42 (37.84%) | 27 (47.37%) | 15 (27.78%) |
| Tx |  | 13 (11.71%) | 1 (1.75%) | 12 (22.22%) |
| **N** |  |  |  |  |
| N1 | / | 47 (42.34%) | 12 (21.05%) | 35 (64.81%) |
| N2-N3 | / | 64 (58.56%) | 45 (78.95%) | 19 (35.19%) |
| **M** |  |  |  |  |
| M0 | / | 21 (18.92%) | 13 (22.81%) | 8 (14.81%) |
| M1 | / | 45 (40.54%) | 30 (52.63%) | 15 (27.78%) |
| Mx | / | 45 (40.54%) | 14 (24.56%) | 31 (57.41%) |
| **Recurrence** |  |  |  |  |
| Yes | / | 1 (0.90%) | 1 (1.75%) | 0 (0%) |
| No | / | 110 (99.10%) | 56 (98.25%) | 54 (100%) |
| **Surgery** |  |  |  |  |
| Yes | / | 48 (43.24%) | 38 (66.67%) | 10 (18.51%) |
| No | / | 63 (56.76%) | 19 (33.33%) | 44 (81.48%) |

**Table S5. IC_50_ of breast cancer, colorectal cancer and pancreatic cancer cell lines in GDSC.**

|  | **Cell** | **EPI (μM)** | **PTX (μM)** | **5-FU (μM)** |
| --- | --- | --- | --- | --- |
| Breast cancer | MDA-468 | 0.290711 | 0.016254 | 20.117204 |
|  | MDB-231 | 0.15131 | 0.085197 | 122.851162 |
|  | MCF-7 | 1.019015 | 0.068046 | 419.464663 |
|  | T47D | 0.431065 | 0.089662 | 23.492153 |
| Colorectal cancer | SW620 | 0.286513 | 0.025688 | 27.195699 |
|  | SW480 | 0.194777 | 0.006887 | 12.492009 |
| Pancreatic cancer | SU86.86 | 4.480252 | 6.478523 | 81.552611 |
|  | SW1990 | 1.191665 | 1.053764 | 38.425613 |
