# Supplementary figures and images for: Chemotherapy induces ACE2 expression in breast cancer via the ROS-AKT-HIF-1α signaling pathway: a potential prognostic marker for breast cancer patients receiving chemotherapy
Source: J Transl Med. 2022 Nov 5;20:509. doi: 10.1186/s12967-022-03716-w (PMC9636712; doi:10.1186/s12967-022-03716-w)

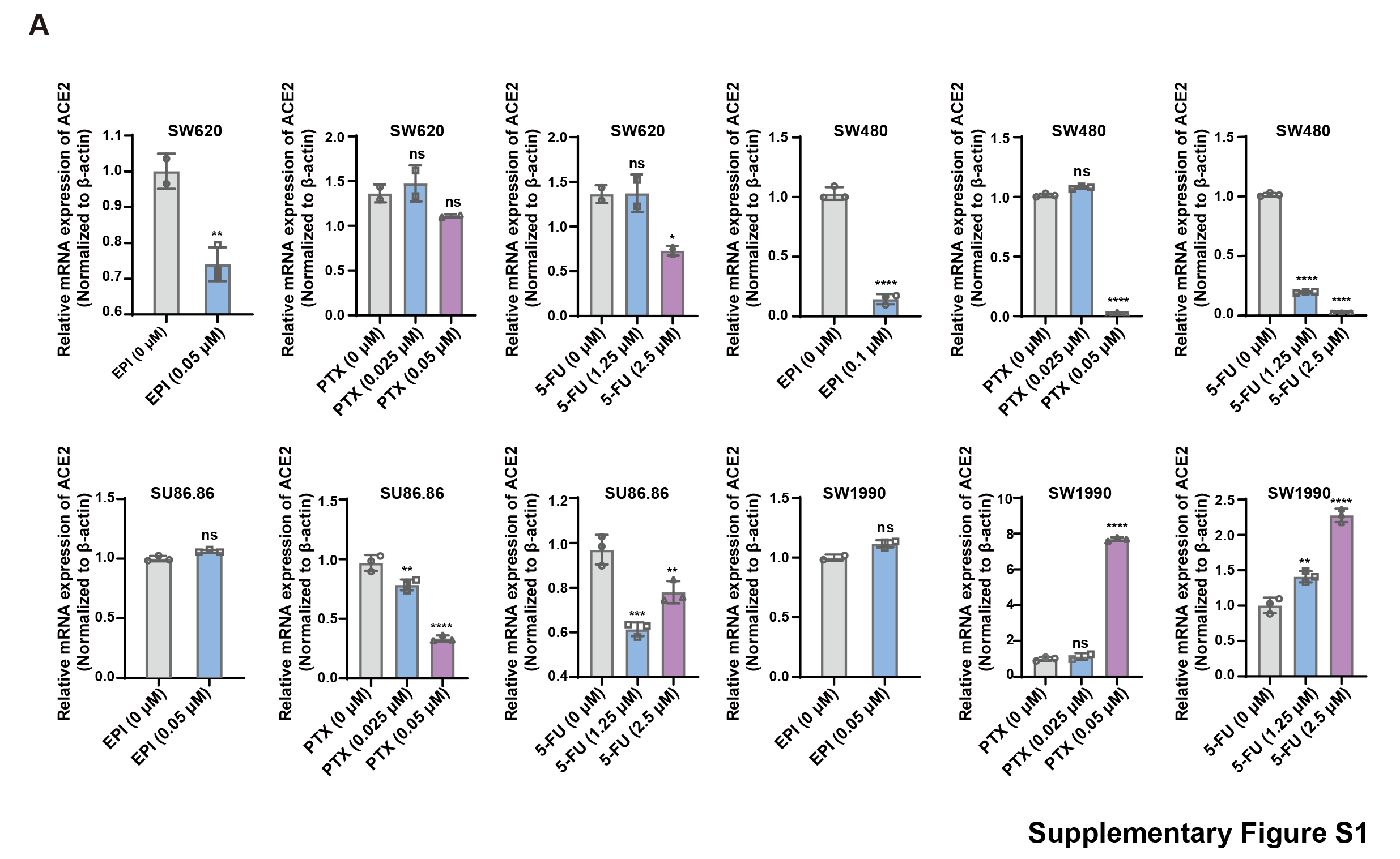

Supplement: Supplementary file 2 — Additional file 2: Figure S1. (A) qRT-PCR analysis showed no significant changes in ACE2 expression in colorectal and pancreatic cancer cells after exposure to EPI, PTX or 5-FU. All data are shown as mean ± SD; *P < 0.05, **P < 0.01, ***P < 0.001, ****P < 0.0001, and ns P > 0.05 versus control, N = 3. [file 12967_2022_3716_MOESM2_ESM.tif]
